# The receptor-like pseudokinase MRH1 interacts with the voltage-gated potassium channel AKT2

**DOI:** 10.1038/srep44611

**Published:** 2017-03-16

**Authors:** Kamil Sklodowski, Janin Riedelsberger, Natalia Raddatz, Gonzalo Riadi, Julio Caballero, Isabelle Chérel, Waltraud Schulze, Alexander Graf, Ingo Dreyer

**Affiliations:** 1 Heisenberg Group of Biophysics and Molecular Plant Biology, Institute of Biochemistry and Biology, Molecular Biology, University of Potsdam, D-14476 Potsdam-Golm, Germany; 2 Max-Planck-Institute of Molecular Plant Physiology, D-14476 Potsdam-Golm, Germany; 3 ETH Zürich, Department of Biology, CH-8092 Zürich, Switzerland; 4 Centro de Bioinformática y Simulación Molecular, Facultad de Ingeniería, Universidad de Talca, Talca, Chile; 5 Plant Biophysics, Centro de Biotecnología y Genómica de Plantas, Universidad Politécnica de Madrid (UPM) - Instituto Nacional de Investigación y Tecnología Agraria y Alimentaria (INIA), E-28223 Pozuelo de Alarcón (Madrid), Spain; 6 Instituto de Biología Vegetal y Fotosíntesis, Consejo Superior de Investigaciones Científicas, E-41092 Sevilla, Spain; 7 Biochimie et Physiologie Moléculaire des Plantes, Centre National de la Recherche Scientifique Unité Mixte de Recherche 5004, Institut National de la Recherche Agronomique U386, Montpellier SupAgro, Université Montpellier II, F-34060 Montpellier cedex 2, France; 8 Department of Plant Systems Biology, University of Hohenheim, D-70593 Stuttgart, Germany

## Abstract

The potassium channel AKT2 plays important roles in phloem loading and unloading. It can operate as inward-rectifying channel that allows H^+^-ATPase-energized K^+^ uptake. Moreover, through reversible post-translational modifications it can also function as an open, K^+^-selective channel, which taps a ‘potassium battery’, providing additional energy for transmembrane transport processes. Knowledge about proteins involved in the regulation of the operational mode of AKT2 is very limited. Here, we employed a large-scale yeast two-hybrid screen in combination with fluorescence tagging and null-allele mutant phenotype analysis and identified the plasma membrane localized receptor-like kinase MRH1/MDIS2 (AT4G18640) as interaction partner of AKT2. The phenotype of the *mrh1-1* knockout plant mirrors that of *akt2* knockout plants in energy limiting conditions. Electrophysiological analyses showed that MRH1/MDIS2 failed to exert any functional regulation on AKT2. Using structural protein modeling approaches, we instead gathered evidence that the putative kinase domain of MRH1/MDIS2 lacks essential sites that are indispensable for a functional kinase suggesting that MRH1/MDIS2 is a pseudokinase. We propose that MRH1/MDIS2 and AKT2 are likely parts of a bigger protein complex. MRH1 might help to recruit other, so far unknown partners, which post-translationally regulate AKT2. Additionally, MRH1 might be involved in the recognition of chemical signals.

The monovalent cation potassium (K^+^) plays important roles in various aspects of the life of plants. It is involved in the regulation of enzyme or transporter activity, protein synthesis, photosynthesis, osmoregulation and phloem transport^1^,^2^. Low potassium availability can lead to decreased resistance to pathogens and plant growth limitation^3^,^4^,^5^,^6^. Potassium is allocated to different plant tissues, organs and cellular compartments by a variety of transporter proteins. The genome of the model plant *Arabidopsis thaliana* encodes at least 71 proteins that are associated with potassium transport^7^. Among them are 9 subunits of tetrameric, voltage-dependent potassium channels. One subunit consists of a shorter cytosolic N-terminus, a membrane spanning core with the voltage-sensor module and the pore module, and a larger cytosolic C-terminus. This C-terminus can encompass up to 65% of the protein and is highly important for channel tetramerization^8^,^9^,^10^ and channel regulation^11^,^12^.

Plant voltage-gated K^+^ channels can be divided into three subfamilies regarding their response to the membrane voltage^13^,^14^. (i) Inward-rectifying (K_in_) channels allow the uptake of K^+^ because they activate upon membrane hyperpolarization and are closed when the driving force for potassium is outwardly directed. (ii) Outward-rectifying (K_out_) channels behave inversely and mediate K^+^ release. (iii) Weak-rectifying (K_weak_) channels can mediate both, K^+^ uptake and release^15^,^16^,^17^,^18^. These channels exhibit unique gating properties and can operate in two different modes. In mode 1 they are K_in_ channels that allow H^+^-ATPase-energized K^+^ uptake, while in mode 2, they are open, K^+^-selective channels^19^. A channel can switch between the two modes via a mechanism that involves reversible phosphorylation affecting the voltage-sensor of the channel^20^,^21^,^22^,^23^. Toggling K_weak_ channels from mode 1 to the voltage-independent mode 2 taps a ‘potassium battery’, providing additional energy for transmembrane transport processes^24^. The battery is charged under energy (ATP) consumption by a hyperpolarizing proton pump and K_in_ channels. The K^+^ ions are then circulated in the phloem and serve as decentralized energy store. This energy can be exploited by regulation of K_weak_ channels to overcome local energy limitations^25^.

AKT2 (AKT2/3) is the only subunit in Arabidopsis that forms K_weak_ channels and became the model for structure-function and physiological studies on this channel type^16^,^17^,^19^,^20^,^21^,^22^,^25^,^26^,^27^,^28^,^29^,^30^,^31^,^32^. Nevertheless, despite these efforts our knowledge on the regulation of AKT2 is still rudimentary. So far, three interaction partners of AKT2 could be identified. One of them is a phosphatase, PP2CA, which converts AKT2 from mode 2 into the inward-rectifying mode^23^. The other two interaction partners, the calcineurin B-like protein CBL4 and the CBL-interacting protein kinase CIPK6 are involved in proper targeting of AKT2 channels to the plasma membrane but do not influence channel activity^30^.

In this study, we screened for other potential interaction partners of AKT2 that might be involved in its functional regulation. We identified the potential leucine-rich repeat receptor-like (LRR) kinase MRH1 (AT4G18640) to interact with AKT2. Null-allele plants for *MRH1* display the same delayed flowering phenotype under energy-limiting conditions that was previously reported for *AKT2* knockout plants. A proof that MRH1 modulates the function or the phosphorylation status of AKT2, however, could not be provided. Instead, a detailed bioinformatics analysis involving structural modeling rather suggests that the kinase domain of MRH1 is not functional. Nevertheless, MRH1 still has the ability of LRR kinases to dimerize. We therefore speculate that MRH1 may recruit another, so far not identified LRR kinase that exerts the modulatory function on AKT2 in the plant.

## Results

### Screening for putative interaction partners of AKT2

**Figure 1:**
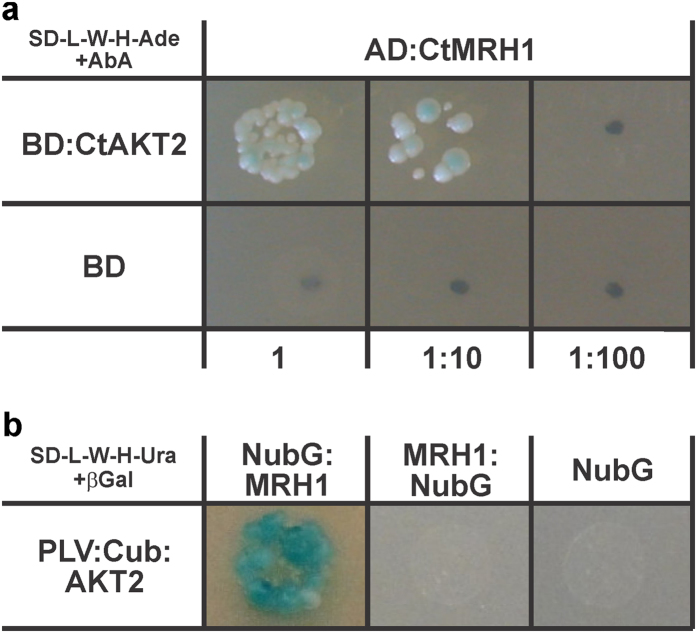
AKT2-MRH1 interaction in yeast. (**a**) Yeast 2-Hybrid interaction test between the cytosolic C-terminal fragment of AKT2 fused to the DNA-binding domain as bait (BD:CtAKT2) and the cytosolic C-terminal fragment of the MRH1 kinase, derived from the cDNA library screening, fused to the activation domain as prey (AD:CtMRH1). Yeast growth on a highly selective medium lacking adenine, histidine, tryptophan, and leucine, supplemented with an antibiotic (AbA), indicate expression of several reporter genes. No growth and thus no reporter gene expression is observed if as bait only the DNA-binding domain (BD) is co-expressed with the AD:CtMRH1 prey. Images were taken after 5 days of growth. 5 μl of mated cultures were plated after dilution and incubated at 30 °C. (**b**) Split Ubiquitin interaction assay between the full-length AKT2 channel fused to the synthetic PLV transcription factor and the C-terminal domain of ubiquitin (PLV:Cub:AKT2 in a bait vector pMetYC-gate) and the full-length MRH1 kinase fused to the modified N-terminal domain of ubiquitin (NubG:MRH1 in prey vector pNX32-GW and MRH1:NubG in prey vector pXN32-GW). Reporter-gene expression can be observed only in one configuration. No auto-activity of the bait was observed. Liquid cultures of the L40 strain harboring both vectors were grown until the mid-logarithmic phase and standardized to OD_600_ = 1.0. Following a 1:10 dilution, 5 μl were plated on a medium containing 5-Brom-4-chlor-3-indoxyl-β-D-galactopyranosid but lacking leucine, tryptophan, adenine and uracil. Images were taken after 7 days of incubation at 30 °C. Results shown in (**a**) were repeated once and in (**b**) are representative for 2 independent repeats.

The Matchmaker Gold Yeast Two-Hybrid system was used to screen a normalized universal Arabidopsis library for proteins that interact with the C-terminally last 484 amino acids of AKT2 (CtAKT2, amino acids 303–787). After two growing steps on highly stringent medium, 81 prey plasmids were isolated and sequenced. Thanks to the normalization of the library, the redundancy of identified preys was rather low. 46 preys were found only once, 9 twice, four were found three times and one prey has been found five times (Table S1). Based on the gene annotation deposited in the Arabidopsis information resource (TAIR, https://www.arabidopsis.org/) we identified a leucine-rich repeat receptor-like kinase (AT4G18640) among the 60 unique preys (Fig. 1a). This kinase is also known as the Morphogenesis of Root Hair formation 1 kinase (MRH1^33^) or MALE DISCOVERER 2 (MDIS2^34^).

### Verification of physical interaction between AKT2 and MRH1

The cDNA identified in the Y2H screen was not a full-length transcript but encoded the C-terminal cytosolic part of MRH1 starting from the middle of the kinase domain (amino-acids 527–678 from 678; Fig. S1). The full-length protein has two predicted membrane domains and is thus not suited for the conventional yeast two-hybrid system that requires cytosolic interaction partners. To verify the interaction of the full-length AKT2/MRH1 pair we used the split ubiquitin-based membrane yeast two-hybrid system. Here, one fusion protein (PLV:Cub:AKT2) comprises the artificial PLV transcription factor, the C-terminal half of yeast ubiquitin (Cub) and full-length AKT2, while the other (NubG:MRH1) is composed of the modified N-terminal half of the ubiquitin moiety (NubG) and the full-length MRH1. Physical association of AKT2 and MRH1 bring the Cub and NubG domains into contact forming a full-length ‘pseudo-ubiquitin’ molecule. This is recognized by cytosolic deubiquitinating enzymes, resulting in cleavage of the transcription factor, and subsequent induction of reporter gene expression (Fig. 1b). The alternative fusion protein MRH1:NubG did not provoke reporter gene expression when co-expressed with PLV:Cub:AKT2, which might indicate that in this combination the NubG and Cub domains are not brought into contact during co-translational protein assembly.

### Co-localization and interaction of AKT2 and MRH1 in plant cells

**Figure 2:**
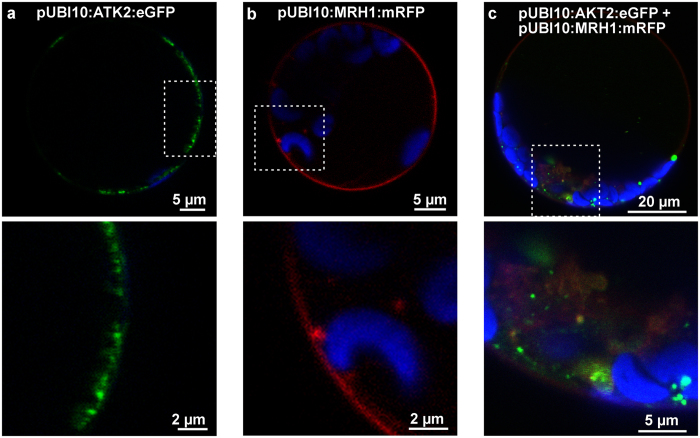
(**a**,**b**) Typical localization of AKT2-eGFP (green) and MRH1-mRFP (red) expressed under the control of the UBI10 promoter and (**c**) co-localization of both in Arabidopsis protoplasts derived from rosette leaves of 3–4 weeks old plants. Blue color corresponds to the auto-fluorescence signal from chlorophyll. Dashed squares indicate zoomed region presented in images below. Images represent single focal planes after 16h–20h post transformation. Shown experiments are representatives of at least 3 independent repeats.

**Figure 3:**
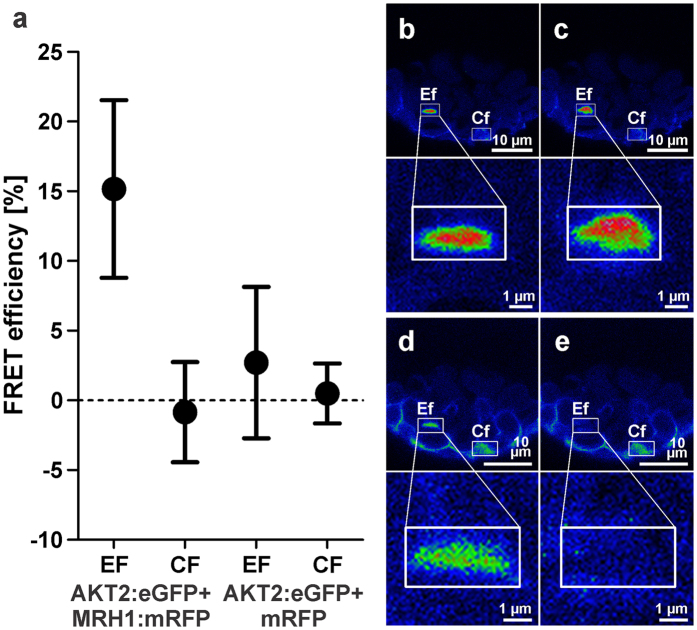
(**a**) FRET measurements in Arabidopsis protoplasts between AKT2:eGFP and MRH1:mRFP or mRFP, only (mean ± SD, n = 10). EF: mean transfer efficiency in a bleached region; CF: mean transfer efficiency in a non-bleached region. For the negative control (AKT2:eGFP and mRFP) no difference between EF and CF values was observed. In contrast, for the AKT2:eGFP + MRH1:mRFP pair the means were significantly different (p > 0.001, Welch two-sample t-test). (**b–e**) Co-expression of AKT2:eGFP and MRH1:mRFP. (**b,c**) Example of an AKT2:eGFP signal before and after bleaching of MRH1:mRFP. (**d,e**) Corresponding regions with signals coming only from MRH1:mRFP before and after bleaching. All experiments were performed on Col0 plants.

To test whether AKT2 and MRH1 physically interact in the native environment, i.e. in plant cells, the channel protein was tagged with eGFP and the receptor-like kinase with mRFP as fluorophores. Both fusion proteins were expressed under the control of the endogenous Arabidopsis ubiquitin 10 promoter (UBI10^35^) in Arabidopsis leaf-derived protoplasts (Fig. 2) and leaves (Fig. S2). Both fusion proteins could be found in the region of the plasma membrane and in intracellular structures resembling the ER. MRH1 and AKT2 signals overlapped, although the relative intensities in the plasma membrane region were lower than in internal structures (Fig. 2; Fig. S2). Encouraged by the observed overlapping localization pattern, interaction between AKT2 and MRH1 was quantified in acceptor photo bleaching Förster resonance energy transfer (FRET) experiments. For FRET detection, the AKT2-eGFP fusion protein was considered as the donor protein and the MRH1-mRFP fusion protein as the acceptor protein. Without photo bleaching of mRFP, the fluorescence of eGFP did not change from one sample interval to the following. The CF-value is therefore zero (Fig. 3). However, after bleaching of the fluorophore in MRH1:mRFP the eGFP emission increased (EF-value > 0) indicating that before part of its excitation energy has been transferred to the acceptor close by. The energy transfer between AKT2:eGFP and MRH1:mRFP indicates that both fusion proteins are in a close proximity. This nearness could emerge from a direct interaction of AKT2 and MRH1 or could be driven by a dimerization of the fluorophores. To discriminate between these alternatives, we repeated the experiment in the absence of MRH1 as proposed by Vermeer *et al*.^36^. When mRFP is not anchored to MRH1, no change in eGFP emission and hence no FRET is observed (Fig. 3a). The appropriateness of the chosen negative control might be argued^37^,^38^; however, the results indicate that mRFP does not stably assemble with AKT2-eGFP excluding a fluorophore-driven proximity of the two fusion proteins.

### Overlapping expression patterns of *AKT2* and *MRH1*

Tissue specific expression of *MRH1* (AT4G18640) and *AKT2* (AT4G22200) was analyzed using BAR^39^, the Bio-Analytic Resource for Plant Biology (http://bar.utoronto.ca/). *MRH1* shows high transcript levels in root hair cells, the root xylem and in the whole stem, in particular in tissues laying below the epidermis. Interestingly, MRH1 expression is elevated upon treatment with ABA in leaf mesophyll and guard cells. Additionally, drought stress has been shown to induce *MRH1* expression in whole rosettes. *AKT2* can be found mainly in the vasculature of stem and roots, specifically in the phloem companion cells^17^. Channel transcripts are also elevated in guard cells upon ABA treatment and upon drought stress in whole rosettes. In summary, although *AKT2* and *MRH1* are assigned to be predominantly active in different tissues, the phloem and root hair cells, respectively, they also share overlapping expression patterns in the above ground tissues and respond to ABA treatment and to drought stress. Thus, both proteins, AKT2 and MRH1, likely have the opportunity to interact in the normal life cycle of a plant.

### *akt2-2* and *mrh1-1* knockout plants are delayed in bolting

**Figure 4:**
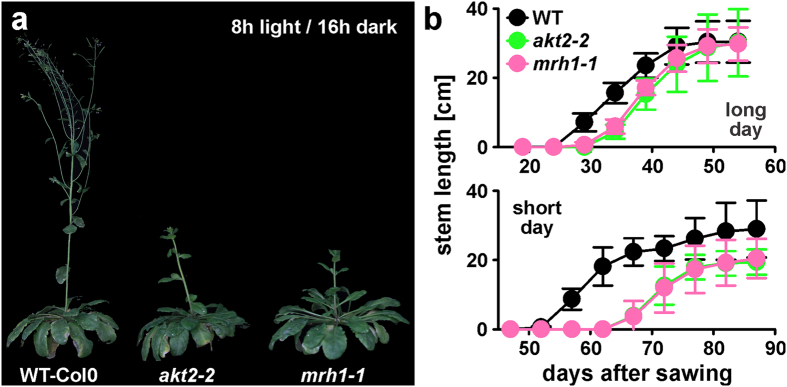
(**a**) Representative image showing *akt2-2, mrh1-1* and WT-Col0 plants grown under short day conditions (8 h light/16 h dark) in a glasshouse 10 weeks after sowing. (**b**) Stem length measurements of all three genotypes grown in a glasshouse in long day (16 h light/8 h dark) and short day conditions (8 h light/16 h dark). Measurements started with the first bolting plant and continued with a 5-day interval until the end of main stalk growth. Data are displayed as mean ± SD (n > 20).

In a previous study it was shown that the loss of AKT2 function provoked a delay in bolting under short day conditions when compared with wild type Wassilewskija plants^25^. In the present study we had to change to the Col0 ecotype because a *MRH1* null-allele plant was available for this ecotype, only^33^. Under short days, the newly isolated *akt2-2* knockout plant in Col0 exhibited the same delayed bolting phenotype as *akt2-1* in Wassilewskija indicating that the bolting phenotype was not ecotype-specific. The same phenotype was observed also for the *mrh1-1* knockout plant (Fig. 4). In Col0 the delay in bolting was not only observable under short day conditions but also in long days, albeit far less pronounced (Fig. 4b). Thus, *akt2-2* and *mrh1-1* have an identical bolting phenotype.

### Lack of evidence for direct functional interaction between MRH1 and AKT2

**Figure 5:**
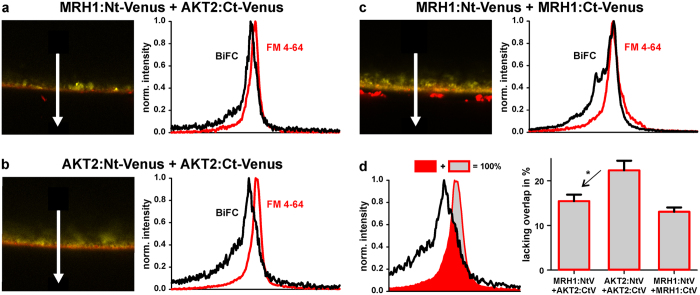
(**a,b,c**) Typical BiFC signals (yellow) recorded for MRH1:Nt-Venus + AKT2:Ct-Venus (**a**), AKT2:Nt-Venus + AKT2:Ct-Venus (**b**), and MRH1:Nt-Venus + MRH1:Ct-Venus (**c**) expressed in *Xenopus leavis* oocytes. Plasma membrane was stained with FM 4-64FX (red) prior imaging. Plots on the right represent signal distribution along the arrows for reconstituted Venus protein (BiFC, black) and the membrane marker (FM 4-64, red). (**d**) Quantitative analysis of the overlap of the BiFC signal with the membrane marker. In comparison to the AKT2:Nt-Venus + AKT2:Ct-Venus pair, the signal overlap of the MRH1:Nt-Venus + AKT2:Ct-Venus pair is significantly larger (P < 0.012, Student´s t-test) indicating an increased integration of the AKT2-protein into the plasma membrane in the presence of MRH1. The signal overlap of the MRH1:Nt-Venus + AKT2:Ct-Venus pair and the MRH1:Nt-Venus + MRH1:Ct-Venus pair is not significantly different. Data shown are representative or are mean ± SE for n ≥ 15.

The results gathered so far point to a functional interaction between MRH1 and AKT2. When analyzing the electrical features of AKT2 after co-expression with MRH1 in *Xenopus* oocytes, however, no effect of MRH1 could be observed. The electrical features of AKT2/MRH1-expressing oocytes were indistinguishable from AKT2-expressing oocytes (Fig. S3). In contrast, the physical interaction of both proteins could be detected in split-YFP bimolecular fluorescence complementation (BiFC) experiments (Fig. 5). As this fluorescence signal could derive not only from the plasma membrane, but also from the endoplasmatic reticulum being very close to the inner surface of the oocyte^40^,^41^, we compared the BiFC signal with the membrane marker FM 4-64FX. This analysis revealed that the presence of MRH1 appeared to facilitate the integration of AKT2 into the plasma membrane. While for the pair AKT2:Ct-Venus + AKT2:Nt-Venus ~22% of the normalized FM 4-64FX and BiFC signals were not overlapping, this value reduced to ~15% upon co-expression of AKT2:Ct-Venus and MRH1:Nt-Venus (Fig. 5d). A similar value (~13%) was also observed for the MRH1:Ct-Venus + MRH1:Nt-Venus pair, indicating that MRH1 can oligomerize similar to other LRR kinases^42^.

These results support functional scaffold features of MRH1 but raised first doubts whether MRH1 is a functional kinase. The doubts were further fueled by *in vitro* phosphorylation assays using the MRH1 kinase domain together with AKT2-derived peptides. In all these experiments a proof of function of the MRH1 kinase domain could not be provided (Table S2).

### MRH1 differs significantly from catalytically active protein kinases

To get further insights into the features of MRH1 (AT4G18640), its kinase domain was compared with those of 10 related kinases from *A. thaliana* (Fig. S4): the inactive kinases ZED1^43^ (AT3G57750), BIR2^44^,^45^ (AT3G28450), RKS1^46^ (AT3G57710), BSK8^47^ (AT5G41260), CORYNE^48^,^49^ (AT5G13290), and STRUBBELIG^50^,^51^ (AT1G11130), as well as the active kinases BRI1^52^ (AT4G39400), BAK1^53^,^54^ (AT4G33430), BIR1^55^ (AT2G31880), and FLS2^56^,^57^ (AT5G46330). This comparison revealed that several conserved kinase motifs^58^,^59^,^60^ are degraded in the kinase domain of MRH1 (Fig. S5). Such loss of conserved motifs has been reported for pseudokinases that contain the structure of a kinase domain but are catalytically inactive^42^,^61^,^62^,^63^. The strongest degradations occur in motif I (G-loop), the last position of motif II (β3 strand) and motif VII (DFG motif). All three motifs fulfill essential functions in kinases. ATP is bound and positioned by the G-loop, the lysine in motif II fixes and arranges the α and β phosphates of the ATP molecule, and the aspartate of the DFG motif chelates the magnesium ion that orients the phosphate for the phosphotransfer^64^,^65^,^66^.

**Figure 6:**
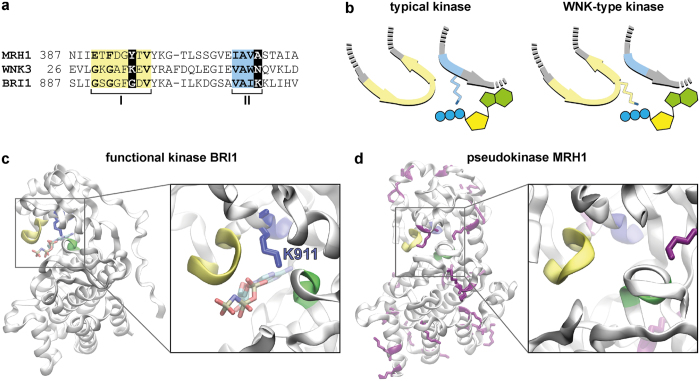
MRH1 has structural properties of a pseudokinase. (**a**,**b**) MRH1 is different from WNK-type kinases that established a strategy for recovery of catalytic activity. (**a**) Sequence comparison of motif I (G-loop) and motif II of MRH1, a typical (BRI1) and a WNK-type kinase (WNK3) from *A. thaliana*. The catalytic lysine in motif II is mutated in MRH1 and WNK3. In WNK-type kinases the third glycine of the G-loop (motif I) is replaced by a lysine. MRH1 does not contain a lysine in neither of the two motifs. (**b**) Recovery strategy of WNK-type kinases. Schematic representation of the catalytic lysine in typical and WNK-type kinases. Motif I is shown in yellow and motif II in blue. In WNK-type kinases the missing catalytic lysine is replaced by a lysine in motif I. (**c**,**d**) Homology model of MRH1 illustrates the absence of coordinating lysine residues in the catalytic center. Three motifs essential for catalytic kinase activity are highlighted in yellow (motif I, G-loop), blue (motif II, β3 strand) and green (motif VII, DFG motif). (**c**) In a typical kinase (BRI1, PDB entry 4OH4) a lysine residue (K911 in BRI1, blue) in motif II coordinates the ATP molecule in the catalytic center (highlighted in licorice representation). (**d**) In MRH1 there is no lysine residue in the vicinity of the potential catalytic center (enlarged view). All 16 lysine residues in the kinase domain of MRH1 are highlighted in purple licorice representation.

Nevertheless, a degenerated kinase motif does not necessarily result in loss of catalytic function^67^,^68^,^69^. A few kinases, as WNK-type kinases for example^70^, developed rescue strategies to compensate the loss of essential amino acids. These kinases miss the catalytic lysine in motif II which is essential for protein kinase enzymatic activity^65^. Instead, the third glycine of motif I (G-loop) is replaced by a lysine that takes over the function of the missing catalytic lysine (Fig. 6a,b). Interestingly, also MRH1 misses the catalytic lysine. However, in contrast to WNK kinases, there is no lysine in the G-loop that may rescue the loss in motif II. A homology model of MRH1 also suggests that no other lysine is present in the ATP binding site capable of replacing the function of the missing catalytic lysine (Fig. 6c,d). Sequence and structure analyses therefore suggest that MRH1 is a pseudokinase; a result that explains well the negative experimental data with respect to kinase activity.

## Discussion

In this study, we identified MRH1 to be a pseudokinase that physically interacts with the weak-rectifying K^+^ channel AKT2. So far, there was no sign of evidence for a connection between both proteins. MRH1 was assigned a role in root hair formation^33^, while AKT2 helps in energizing phloem (un-)loading processes^25^. Initially, we were searching for a kinase that modifies the phosphorylation status of AKT2, which switches the channel from an inward-rectifier into an open, K^+^-selective channel. This in turn taps a ‘potassium battery’, providing additional energy for transmembrane transport processes. But, at the functional level, no effect of MRH1 on the activity of AKT2 could be observed in heterologous expression systems. Knockout plants that lack the *MRH1* gene, however, mirror the phenotype of *AKT2* knockout plants or that of plants, in which AKT2 cannot be converted anymore into a non-rectifying channel^22^,^25^. Thus, MRH1 might be involved *in vivo* in the same task as the open AKT2, *i.e.* tapping the ‘potassium battery’. How could MRH1 exert this function in a kinase activity-independent manner? Sequence and structure analyses suggest that MRH1 is a pseudokinase. Despite the lacking kinase activity, it still has functional scaffold properties. Besides interacting with AKT2, it also forms oligomeric complexes with itself and with its twin MRH2/MDIS1^34^ (BiFC data from this study not shown). These data suggest that MRH1 behaves as normal receptor kinases that form functional hetero-oligomeric complexes involving other receptor kinases and target proteins^62^,^71^,^72^,^73^,^74^. Within these complexes not all proteins need to interact with each other. In the PSRK1/BAK1/AHA1/AHA2/CNGC17-complex, for instance, the leucine-rich repeat receptor kinase PSKR1 interacts with the plasma membrane-localized H^+^-ATPases AHA1 and AHA2 and with the receptor kinase BAK1, while CNGC17 interacts with AHA1, AHA2, and BAK1 but not directly with PSRK1^75^. Thus in analogy, AKT2 and MRH1 might be entities of larger complexes, and MRH1 might help to recruit other, so far unknown partners, which post-translationally regulate AKT2. In addition, the receptor pseudokinase MRH1 might also be involved in the reception of so far unknown signaling molecules. Within a larger protein complex the binding of the signal molecule to MRH1 could induce diverse conformational changes not only in MRH1 but also in connected proteins that then modify the activity of other kinases that finally modulate AKT2. Larger protein complexes with different receptors seem reasonable as they would allow a fine-tuned regulation of the AKT2 channel in response to diverse signals.

## Methods

### Yeast Two-Hybrid screens

Matchmaker Gold yeast 2-hybrid system with bait vector pGBKT7 and prey vector pGADT7 (Takara/Clontech) was employed to screen the ‘Normalized Universal Arabidopsis Mate & Plate Library’ (Takara/Clontech) using the C-terminal fragment of AKT2 (CtAKT2, amino acids 303–787) as bait. Library screening and preceding tests were performed according to the manufacturer’s manual. After mating of bait and library strains, the entire cell mixture was plated on 5000 cm^2^ of low stringency selection medium (SD-L-W, supplemented with 1 mg/ml X-β-Gal and 50 μM aureobasidin A) and kept for 5 days at 30 °C. Emerging blue and white colonies were amplified separately overnight in SD-L-W liquid medium, washed and re-suspended in 1 ml of 1 M sorbitol. 5 μl of the dilution mixture was dropped on solid medium of higher stringency (SD-L-W-H-Ade, supplemented with 1 mg/ml X-β-Gal and 50 μM aureobasidin A) and kept at 30 °C. Colonies appearing within 3, 5 and 7 days were collected and the prey vector was isolated, amplified and sequenced. For independent verification, an appropriate yeast strain was transformed with the bait construct and a verified prey clone and submitted for direct growth tests and reporter gene expression analyses on high stringency solid medium.

### Interaction tests using the Yeast Split Ubiquitin system

The basis of the split ubiquitin system was as described in Obrdlik *et al*.^76^. Baits were cloned into pMetYC-Gate and preys into pX-NubWT-Gate or pNubWT-X-Gate. Because of large auto-activation and instability, the strains THY.AP4 and THY.AP5 were replaced by the strain L40^8^ and instead of mating, we used co-transformation. 10–15 co-transformed, freshly emerging yeast colonies were inoculated in 5 ml of liquid SD-L-W media selective for both vectors and grown over-night at 30 °C. Saturated cultures were diluted 10 times and grown until the mid-log phase in the same conditions. Finally, an aliquot was re-suspended in 1 M sorbitol to achieve the same cell density (OD_600_ of 1). These normalized cultures were diluted 10 times. 5 μl of this dilution were dropped on solid selective SD-L-W-U-H medium. In addition, 3-AT was supplied to increase the selection pressure. X-β-Gal was added along with BU salts to visualize the β-galactosidase activity. After drying, plates were incubated at 30 °C and images of growing colonies were taken after 7 days.

### BiFC in *Xenopus* oocytes

Expression of proteins for BiFC experiments in *Xenopus* oocytes was carried out as described^77^. Imaging of oocytes was performed on Leica TCS-SP8 confocal system. Samples were analyzed using HC PL APO 40x/1.10 W objective. Samples were stored in a modified Barth’s medium^78^ and were incubated with FM 4-64FX (Invitrogen) prior measurement at a concentration of 10 μg/ml for 5 min. After washing, samples were imaged using 514 nm argon and 561 nm diode lasers. Emission light was separated by AOBS and collected using HyD detectors at 525–575 nm for BiFC signal and 576–626 nm for FM 4-64FX signal. Images were always acquired with resolution of ~100 nm/pixel in an 8-bit mode. Imaging conditions between samples and experiments were not changed except for the MRH1:NtV + MRH1:CtV pair, where the gain value of the detector was adjusted to fit the dynamic range. For each BiFC combination at least 3 individual cells were used for imaging. Data from each recorded single plain image was collected at five different regions and used for signal overlay analysis.

### Localization in Arabidopsis protoplasts and leaves

Protoplasts from 3–4 weeks old Arabidopsis rosette leaves were isolated following a procedure adapted from Wu *et al*.^79^. The abaxial site of the leaf epidermis was peeled off using common transparent office tape. Fragments from 10 leaves with removed epidermis were placed in 10 ml of an enzyme solution and incubated for 1 h at RT with gentle agitation. Protoplasts were released from digested leaves by increased agitation for additional 10–15 min of incubation. Protoplasts were collected by centrifugation at 100 g for 3 min at 4 °C and re-suspended in 7.5 ml of ice cold W5 solution (154 mM NaCl, 125 mM CaCl_2_, 5 mM KCl, 2 mM MES, 5 mM Glucose). Following another washing step, the protoplasts were left on ice for 30 min. Thereafter, protoplasts were collected by centrifugation at 100 g for 3 min at 4 °C and re-suspended in 2 ml of ice cold MMg solution (0.4 M Mannitol, MES 4 mM, 15 mM MgCl_2_). Transformation was carried out at RT by mixing 10–15 μg of DNA per construct with 200 μl of the protoplast solution and equal volume of the PEG solution (40% (w/v) PEG 4000, 0.2 M Mannitol, 0.1 mM CaCl_2_). The mix was left for 10–15 min. and then washed with W5 solution 3 times at room temperature. Each time, protoplasts were collected by centrifugation at 100 g for 2 min at RT. Finally, transformed protoplasts were re-suspended in 0.5 ml W5 solution and incubated in the dark at RT for 16h–20h.

Transient expression *in planta* was performed using biolistics. Gold particles of 0.6 μm radius were coated with appropriate DNA constructs. 25 μl of gold particles (60 mg/ml in H_2_O) were mixed with 10 μg of DNA re-suspended in less than 5 μl volume on ice. The mixture was vortexed briefly and 25 μl of 2 M CaCl_2_ was added. Samples were vortexed again and 20 μl of 0.1 M spermidine were added and centrifuged at 4 °C at 13,000 g. 250 μl of absolute EtOH were added and vortexed thoroughly for 1 min followed by centrifugation at 13,000 g at 4 °C. Collected DNA-coated particles were then washed again with 400 μl absolute EtOH and re-suspended in 20–30 μl absolute EtOH. Rapture disks, micro-carriers and stopping screens were rinsed in absolute EtOH and dried. DNA-coated particles for each construct were transferred onto a rapture disk and kept at RT until dried. All elements were then assembled for single shots using the PDS-1000 biolistic particle delivery system in accordance to the manufacturer’s guidelines (Bio-Rad). At least three adult rosette leaves from 4–5 weeks old Arabidopsis plants were placed on a Petrie dish with their abaxial side facing the particle system outlet. After bombardment, leaves were rinsed with deionized water, placed on a filter paper (Munktell & Filtrak, Bärenstein, Germany), soaked with water and sealed with tape (Leukopor, Hamburg, Germany). All samples were than kept vertically in the dark for at least 48 h at RT prior analysis.

Imaging was performed on a Leica SP5 confocal microscope in a sequential mode equipped with HC PL APO 63x/1.20 W CORR CS2 objective. eGFP constructs were excited using a 488 nm argon laser, mRFP using a 561 nm diode laser. Emitted light was filtered using AOBS and collected by HyD detectors (500–525 nm for eGFP and 576–501 nm for mRFP). Pinhole was set to 1 Airy unit. Detector amplification and laser power were adjusted for the best signal to noise ratio.

### FRET

FRET experiments were performed according to Karpova *et al*.^80^ in a sequential mode on the Zeiss LSM 710 inverted system with plan-apochromat 63x/1.40 Oil DIC M27 oil immersion objective. 488 nm argon laser was used to excite the donor and 561 nm diode laser was used to excite the acceptor proteins. Emission light was collected using an appropriate filter set with PMT detectors. The pinhole was set to 1 Airy unit. Imaging was performed in sequential mode on regions directly adhering to the cover slip. For each region of interest, time lapse of 15 frames was done with an interval smaller than 1 s. Bleaching of acceptor was induced after the fifth frame. Images were taken in an 8-bit mode with 4.5 zoom at 352 × 353 pixels giving a final resolution of ∼85 nm\pixel.

FRET efficiency (Ef) and false FRET (Cf, which always refers to a non-bleached region) were calculated according to the equation 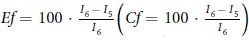, where *I*_*5*_ and *I*_*6*_ denote the intensity of a frame 5 and 6 recorded for the donor channel in a specified region.

### Plant cultivation and phenotypic analyses

Arabidopsis seeds from SALK lines SALK_004879 (*mrh1-1*) and SALK_017212 (*akt2-2*) were ordered from NASC (http://arabidopsis.info/BasicForm). Seeds were verified for homozygous insertion by PCR reaction using primers and conditions described^33^. Col-0 seeds were received from in-house stocks of Institute of Biochemistry and Biology (University of Potsdam, Germany). Prior sowing, all seeds were kept for 3 days in the dark at 4 °C. Temperature in the glasshouse was kept at 21 °C during the day and 19 °C during the night. Plants were grown in the third and fourth quarter of the year. In long day-conditions lights were switched on at 6 a.m. and switched off at 10 p.m. In short-day conditions lights were switched on at 8 a.m. and switched off at 4 p.m. Stem length was measured every 5 days starting with the first bolting plant. At least 20 plants of each genotype were used for characterization.

### Bioinformatic analyses of the MRH1 protein

The 678 amino acids long MRH1 protein was analyzed using the Conserved Domain Database (CDD)^81^ to identify characteristic protein domains and TMHMM^82^ to predict transmembrane spanning segments. These searches suggest a Leucine Rich Repeat (LRR) N-terminal domain between positions 26 and 67, an LRR domain between 96 and 154, a protein kinase domain between positions 396 and 649, and two transmembrane regions between positions 10 and 32 and between 320 and 342 (Fig. S1).

Multiple sequence alignment of MRH1 in comparison to active and inactive protein kinases from *Arabidopsis thaliana* was performed with T-Coffee^83^ and examined using Jalview^84^. Sequence Logos were produced using WebLogo3.4^85^. A structural model of MHR1’s putative kinase domain was created with the I-Tasser server^86^. I-Tasser first generates full-length atomic structural models from multiple threading alignments and iterative structural assembly simulations followed by atomic-level structure refinement. As threading templates served the *Arabidopsis thaliana* pseudokinase BIR2 (4L68), the kinases BRI1 (4OH4) and BAK1 (3UIM) and the human IRAK4 kinase (2OIB).

### Electrophysiology

Experiments on *Xenopus* oocytes were carried out as described previously^19^,^20^,^21^,^78^.

### Phosphorylation analysis

Short peptides corresponding to amino acids 200–222 and 319–341 from AKT2 and the whole predicted cytosolic fragment of MRH1 kinase (amino acids 344–678) were cloned into pPICZαA (Invitrogen) expression vector. Confirmed clones were transformed with EasyComp Transformation Kit (Thermo-Fisher) into *P. pastoris* strain SMD1168H (Invitrogen). Growth of positive colonies expressing and secreting recombinant proteins was performed in accordance to the Pichia Expression Kit (Thermo-Fisher). Expression for each colony was tested by a dot-blot or a SDS-PAGE followed by a Western blot against c-Myc-tag antigen. Detection was performed on LI-COR Odyssey Imaging System using respective IRDye800CW secondary antibody (LI-COR). Final cultures were scaled up to 100 ml and induced every 24 h with 0.5% (w/v) methanol, finally a supernatant was collected for purification with Protino Ni-RDA resin (Macherey-Nagel) against C-terminally located 6xHis-tag. Samples were incubated with the resin and later eluted according to the manufacturer’s instructions at 4 °C. Protein concentration was measured using NanoOrange Protein Quantitation Kit (Thermo-Fisher). The subsequent phosphorylation assay was carried out with and without the addition of TritonX-100 as described^87^,^88^. A molar ratio of 1:1 between kinase cytosolic fragment and the AKT2 derived peptides was set and the final ATP concentration was 800 nM. Reactions were prepared on ice followed by 1 h at 25 °C. To stop the reaction, acetone was added. For precipitation, samples were left overnight at −20 °C. In-solution digest with sequencing grade modified trypsin was performed as described^89^ prior direct analysis on the whole mixture which included AKT2 peptides and the kinase fragment without phosphopeptide enrichment. Tryptic peptides were analyzed by LC−MS/MS system using nanoflow Easy-nLC coupled with an LTQ-Orbitrap hybrid mass spectrometer (Thermo Scientific). Samples were injected to the LTQ-Orbitrap using a linear acetonitrile gradient ranging from 5% to 65% within 90 min. Up to 5 MS/MS scans were acquired at a 60,000 FWHM with a cycle time of 1 s. Proteins were identified using MaxQuant version 1.4.2.1 with Andromeda search engine. All spectra were matched against the Arabidopsis proteome (TAIR10). Trypsin and keratin were set as contaminants, carbamidomethylation of cysteine was set as a fixed modification, oxidation of methionine and phosphorylation of serine, threonine, and tyrosine were set as variable modifications during the database search. Two missed cleavages were allowed and mass tolerance for the database search was set to 10 ppm on full scans and 0.5 Da for fragmentations. Multiplicity was set to 1. The obtained peptide list was first searched for the presence of kinase, and AKT2 peptides using cRacker^90^. Thereafter, the list was inspected manually for the presence of AKT2 peptides with selected variable phosphorylation at amino acids S, T and Y.

## Additional Information

**How to cite this article**: Sklodowski, K. *et al*. The receptor-like pseudokinase MRH1 interacts with the voltage-gated potassium channel AKT2. *Sci. Rep.* **7**, 44611; doi: 10.1038/srep44611 (2017).

**Publisher's note:** Springer Nature remains neutral with regard to jurisdictional claims in published maps and institutional affiliations.

## Supplementary Material

Supplementary Material
