# Supplementary material for: The receptor-like pseudokinase MRH1 interacts with the voltage-gated potassium channel AKT2
Source: Sci Rep. 2017 Mar 16;7:44611. doi: 10.1038/srep44611 (PMC5353636; doi:10.1038/srep44611)
Supplement: Supplementary Material [file srep44611-s1.doc]

**The receptor-like pseudokinase MRH1 interacts with the voltage-gated potassium channel AKT2**

Kamil Sklodowski, Janin Riedelsberger, Natalia Raddatz, Gonzalo Riadi, Julio Caballero, Isabelle Chérel, Waltraud Schulze, Alexander Graf, Ingo Dreyer

Supplementary Material

**Figure S1**


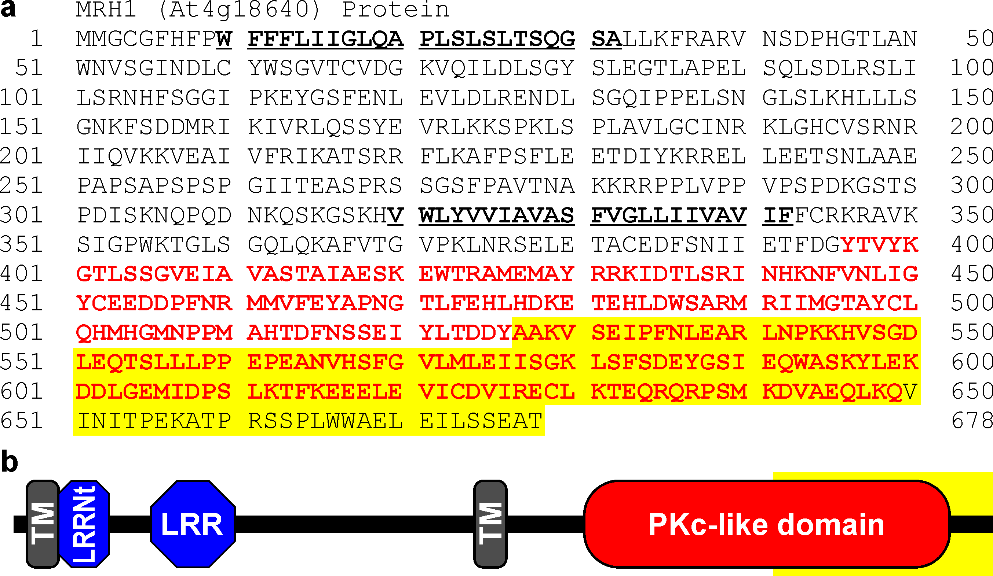


**Figure S1.** Features of the protein MRH1 (AT4G18640). (**a**) Protein sequence of MRH1 with the predicted transmembrane domains underlined and the kinase domain in red. The fragment indicated in yellow (amino acids 527-678) was identified in the yeast two-hybrid screen. (**b**) Visualization of predicted domains based on CDD search and transmembrane regions (TM). Two TM regions are predicted between positions 10 and 32 and positions 320 and 342 (*grey*). A Leucine Rich Repeat (LRR) domain and its N-terminal domain (LRRNt) are predicted between positions 96 and 154 and positions 26 and 67 (*blue*) as well as a kinase domain between positions 396 and 649 (*red*).

**Figure S2**


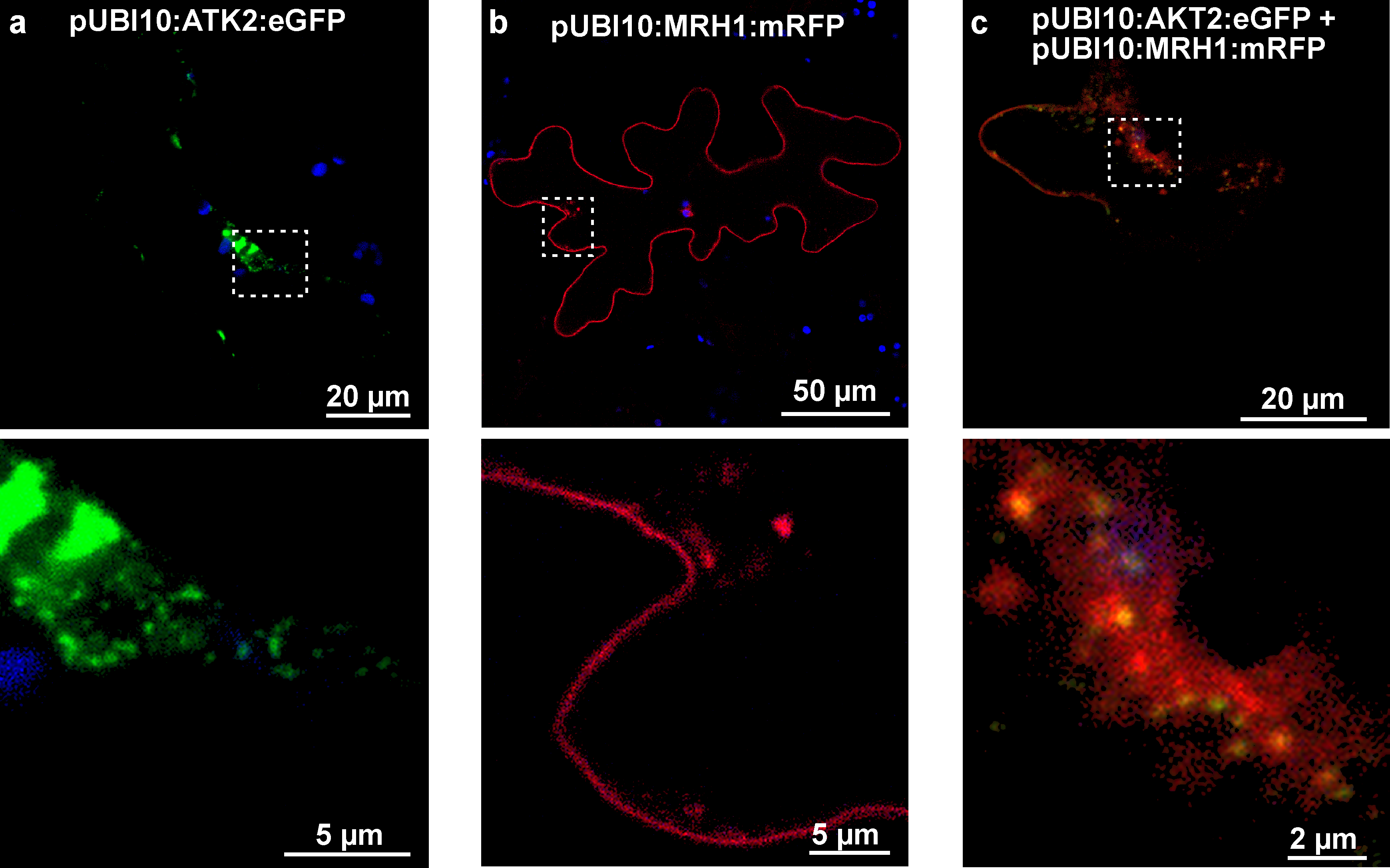


**Figure S2.** (**a, b**) Typical localization of AKT2:eGFP (green) and MRH1:mRFP (red) expressed under the control of the UBI10 promoter and (**c**) co-localization of both in Arabidopsis rosette leaves of 4-5 weeks old plants after particle bombardment. Blue color corresponds to the auto-fluorescence signal from chlorophyll. Dashed squares indicate zoomed region shown in adjacent images. Images represent single focal planes after 48h-60h post transformation. Experiments were performed on Col0 plants and are representative for at least 3 independent repeats.

**Figure S3**


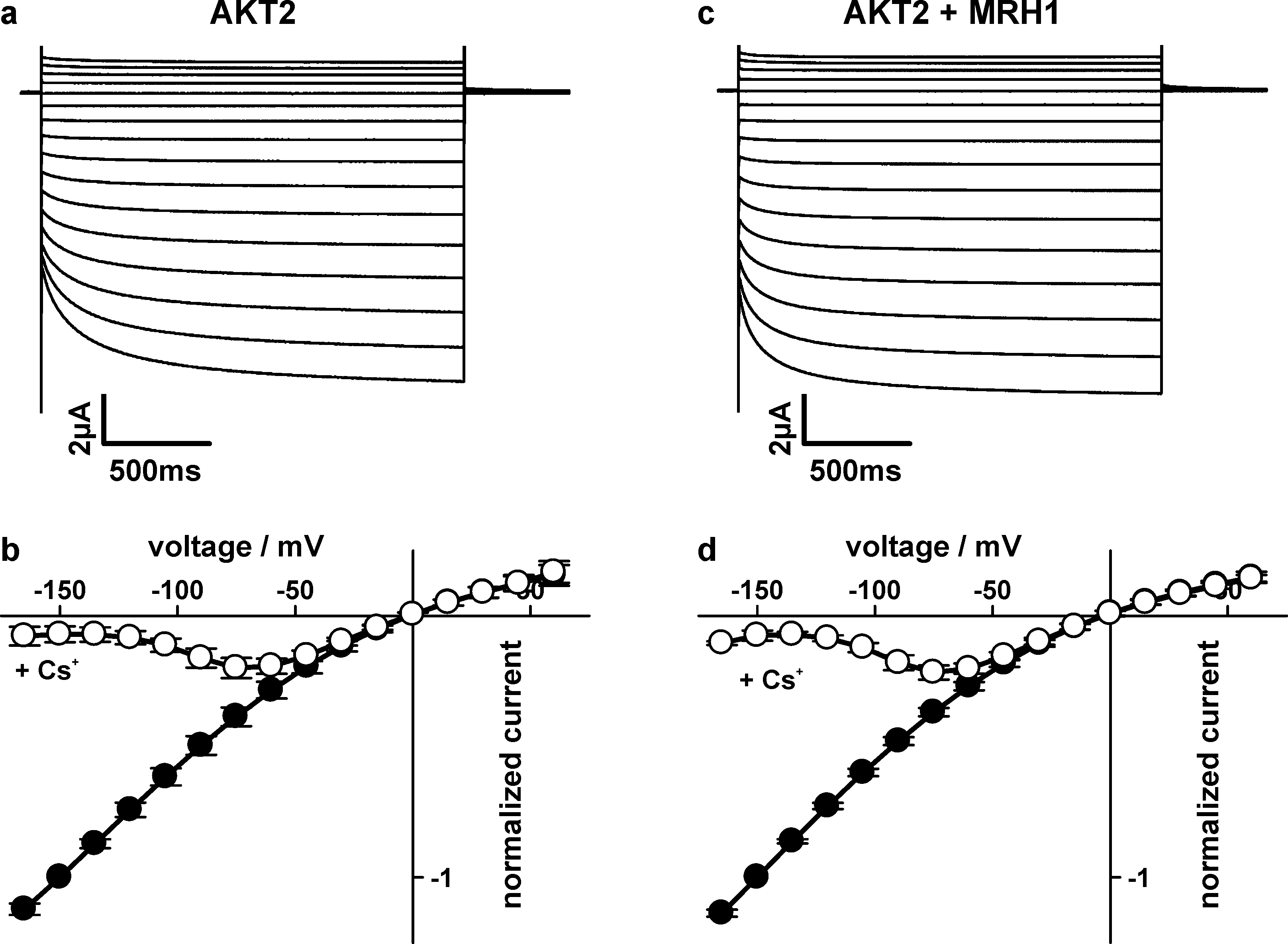


**Figure S3.** MRH1 does not affect the electrical features of AKT2 upon co-expression in *Xenopus* oocytes. (**a**, **b**) K^+^ currents measured in AKT2-expressing oocytes. (**c**, **d**) K^+^ currents measured in oocytes co-expressing AKT2 and MRH1. (**a**, **c**) Representative current traces elicited by 2-s voltage pulses to voltages ranging from +60 mV to -165 mV (15-mV intervals) starting from a holding voltage of 0 mV. (**b**, **d**) Current-voltage characteristics of the currents measured at the end of the activating voltage pulses. Data are mean±SD of 7 (AKT2) and 5 (AKT2+MRH1) independent experiments. The external standard solution was composed of 100 mM KCI, 2 mM MgCl_2_, 1 mM CaCl_2_, and 10 mM Hepes-Tris, pH 7.4 (black symbols). To distinguish K^+^ currents and any background, non-selective leak, measurements were repeated routinely with the addition of 10 mM CsCl (white symbols).

**Figure S4**


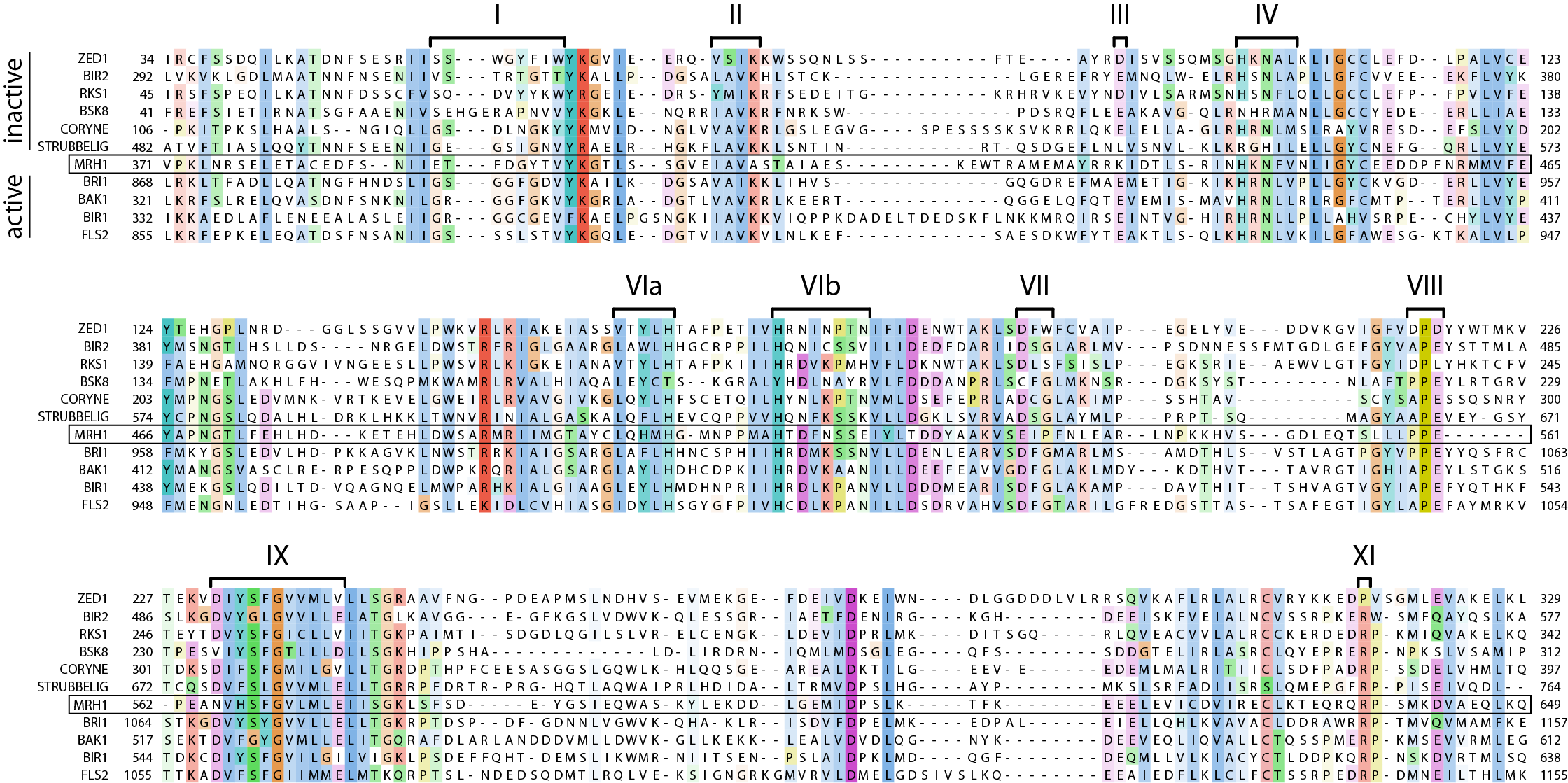


**Figur****e S4.** Multiple sequence alignment of MRH1 and active and inactive kinases from *A. thaliana*. The conversed kinase motifs shown in **Fig. S5** are marked. Residues are colored according to the Clustalx coloring mode.

**Figure S5**


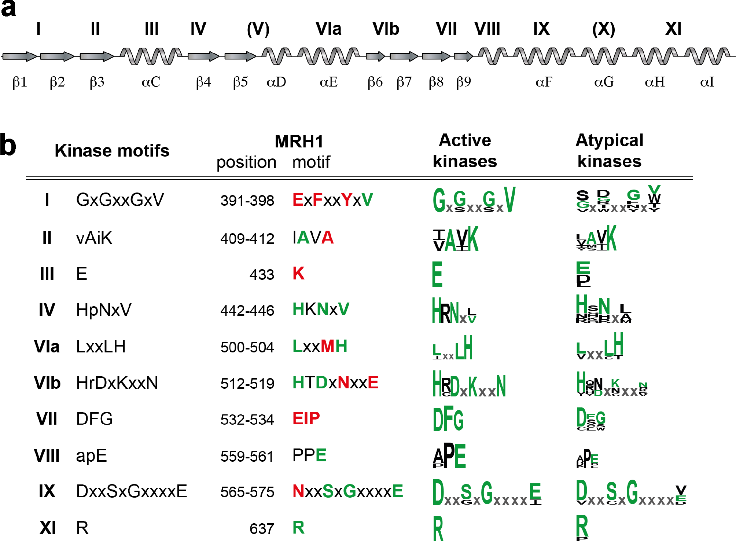


**Figure S5.** Conservation of kinase motifs of active and pseudokinases from *Arabidopsis thaliana* in comparison to MRH1. The presence of motifs conserved in functional kinases has been examined in MRH1 in comparison to a set of active and pseudokinases from *A. thaliana*. (**a**) Schematic representation of the location of conserved motifs (I-XI) along the kinase structure (α−helices and β−sheets indicated). (**b**) Variations of conserved motifs are marked in red and conservations in green for MRH1. Conservation of motifs in active and pseudokinases is illustrated in form of sequence logos.

**Table S1** Putative AKT2-interacting partners identified in the yeast two-hybrid screen

| **AGI locus code** | **times found in screen** | **Annotation** | **Predicted subcellular localization** |
| --- | --- | --- | --- |
| ATCG00065 | 1 | Ribosomal protein S12 | chloroplast; encoded by chloroplast DNA |
| AT5G55220 | 1 | trigger factor type chaperone family protein | chloroplast, chloroplast envelope, chloroplast stroma |
| AT5G52550 | 1 | hypothetical protein | unknown |
| AT5G50750 | 1 | reversibly glycosylated polypeptide 4 | Golgi apparatus, cell junction, cytosol, plant-type cell wall |
| AT5G40950 | 1 | ribosomal protein large subunit 27; RPL27 | chloroplast, chloroplast envelope, chloroplast stroma, ribosome, thylakoid |
| AT5G15280 | 1 | pentatricopeptide repeat-containing protein | unknown |
| AT5G13420 | 1 | transaldolase | chloroplast, chloroplast stroma, mitochondrion |
| AT5G09800 | 1 | U-box domain-containing protein 28 | cytosolic, as it is related with E3 class ubiquitin ligases |
| AT5G05240 | 1 | hypothetical protein | unknown |
| AT4G38620 | 1 | MYB4 | Nuclear localization of MYB4 depends on the action of the beta importin SAD2 |
| AT4G32040 | 1 | homeobox protein knotted-1-like 5 | nucleus |
| AT4G30350 | 1 | heat shock-related protein | unknown |
| AT4G18640 | 1 | putative LRR receptor-like serine/threonine-protein kinase MRH1 | unknown |
| AT4G17730 | 1 | SYP23 syntaxin-23 | intracellular, vacuolar membrane |
| AT4G12700 | 1 | hypothetical protein | unknown |
| AT4G11260 | 1 | phosphatase SGT1b | SCF ubiquitin ligase complex, cytoplasm, cytosol, nucleus |
| AT4G11010 | 1 | nucleoside diphosphate kinase 3 | inter-membrane space in mitochondria |
| AT3G55650 | 1 | pyruvate kinase | mitochondrion |
| AT3G54826 | 1 | Zim17-type zinc finger protein | unknown |
| AT3G25530 | 1 | glyoxylate reductase 1 | cytosolic |
| AT3G24630 | 1 | hypothetical protein | cellular component |
| AT3G15950 | 1 | DNA topoisomerase-like protein | ER body, membrane, peroxisome, plasmodesma |
| AT3G12980 | 1 | histone acetyltransferase HAC5 | nucelus |
| AT3G02950 | 1 | Tho complex subunit 7/Mft1p | unknown |
| AT3G02550 | 1 | LOB domain-containing protein 41 | unknown |
| AT2G42790 | 1 | citrate synthase 3 | peroxisome |
| AT2G42270 | 1 | U5 small nuclear ribonucleoprotein helicase | plasmodesma |
| AT2G41520 | 1 | DNAJ heat shock N-terminal domain-containing protein | unknown |
| AT2G41250 | 1 | haloacid dehalogenase-like hydrolase domain-containing protein | unknown |
| AT2G37940 | 1 | inositol phosphorylceramide synthase 2 | unknown |
| AT2G37500 | 1 | arginine biosynthesis bifunctional protein ArgJ | chloroplast, chloroplast stroma |
| AT2G36530 | 1 | bifunctional enolase 2/transcriptional activator | apoplast, chloroplast, cytoplasm, cytosol, membrane, mitochondrial envelope, mitochondrion, nucleus, plasma membrane, plasmodesma |
| AT2G33430 | 1 | DAL1 | chloroplasts |
| AT1G70320 | 1 | ubiquitin-protein ligase 2 | cytosol, mitochondrion, nucleus, ubiquitin ligase complex, not located in cytosol |
| AT1G63670 | 1 | hypothetical protein | unknown |
| AT1G58070 | 1 | hypothetical protein | unknown |
| AT1G56280 | 1 | drought-induced 19 protein | nuclear localization signal |
| AT1G56020 | 1 | hypothetical protein | unknown |
| AT1G51510 | 1 | RNA-binding protein 8A | cytoplasm, exon-exon junction complex, nuclear body, nucleolus, nucleoplasm |
| AT1G34355 | 1 | forkhead-associated (FHA) domain-containing protein; PS1 | unknown |
| AT1G28290 | 1 | arabinogalactan protein 31; AGP31 | plasma membrane, plasmodesma |
| AT1G19180 | 1 | protein TIFY 10A/JAZ1 | nucleus |
| AT1G12330 | 1 | hypothetical protein | chloroplast |
| AT1G11930 | 1 | putative pyridoxal phosphate-dependent enzyme, YBL036C type | unknown |
| AT1G06900 | 1 | putative N-arginine dibasic convertase | cytosolic |
| AT1G02680 | 1 | transcription initiation factor TFIID subunit D11 | transcription factor complex, nuclei? |
| AT5G51850 | 2 | hypothetical protein | unknown |

| AT3G45140 | 2 | lipoxygenase 2 | chloroplast, chloroplast envelope, chloroplast stroma, chloroplast thylakoid membrane, cytoplasm |
| --- | --- | --- | --- |
| AT3G04120 | 2 | glyceraldehyde-3-phosphate dehydrogenase, cytosolic | apoplast, chloroplast, cytosol, membrane, mitochondrial envelope, mitochondrion, nucleus, plasma membrane, vacuolar membrane |
| AT1G80620 | 2 | S15/NS1, RNA-binding protein | chloroplast, intracellular, ribosome, small ribosomal subunit |
| AT1G74160 | 2 | hypothetical protein | plasmodesma |
| AT1G62780 | 2 | hypothetical protein | unknown |
| AT1G16710 | 2 | histone acetyltransferase HAC12 | nucleus |
| AT1G13635 | 2 | DNA-3-methyladenine glycosylase I | unknown |
| AT1G07120 | 2 | hypothetical protein | chloroplast envelope |
| AT4G34430 | 3 | SWI/SNF complex subunit SWI3D | unknown |
| AT4G32530 | 3 | ATPase, F0/V0 complex, subunit C protein | vacuole |
| AT2G26460 | 3 | cytokine | nucleus |
| AT1G35720 | 3 | annexin D1 | apoplast, cell wall, chloroplast, chloroplast stroma, cytosol, membrane, mitochondrion, plasma membrane, plasmodesma, thylakoid, vacuolar membrane, vacuole |
| AT5G40590 | 5 | cysteine/histidine-rich C1 domain-containing protein | cellular component |

**Table S2.** Detected AKT2 peptides with their sequences and intensities after protein identification using MaxQuant. As described in the methods section, AKT2 peptides (amino acids 200-222 and 319-341) were preincubated with the whole predicted cytosolic fragment of MRH1 (amino acids 344-678). No modifications of AKT2 peptides were found with and without addition of Triton. Crucial serine residues 210 and 329 of the AKT2 protein are marked in red.

| **Fasta headers** | **Sequence** | **Intensity** | **Score** | **PEP** | **Modifications** | **Additives in reaction** |
| --- | --- | --- | --- | --- | --- | --- |
| AT4G18640 | AMEMAYR | 562060 | 82.305 | 0.037232 | Unmodified | none |
| AT4G18640 | DDLGEMIDPSLK | 534910 | 82.85 | 0.0067131 | Oxidation (M) | none |
| AT4G18640 | DDLGEMIDPSLK | 1056100 | 53.448 | 0.043228 | Oxidation (M) | none |
| AT4G18640 | DDLGEMIDPSLK | 1764900 | 135.1 | 0.0012987 | Unmodified | none |
| AT4G18640 | DDLGEMIDPSLK | 4011000 | 113.61 | 0.0030937 | Unmodified | none |
| AT4G18640 | DVAEQLKQVINITPEK | 1672400 | 109.67 | 0.00047583 | Unmodified | none |
| AT4G18640 | DVAEQLKQVINITPEK | 1285100 | 131.56 | 0.00017918 | Unmodified | none |
| AT4G18640 | DVAEQLKQVINITPEK | 6475600 | 131.56 | 0.00017918 | Unmodified | none |
| AT4G18640 | DVAEQLKQVINITPEK | 6338100 | 127.3 | 0.00018258 | Unmodified | none |
| AT4G18640 | ETEHLDWSAR | 1232600 | 81.017 | 0.033693 | Unmodified | none |
| AT4G18640 | GTLSSGVEIAVASTAIAESK | 155360 | 58.123 | 0.045189 | Unmodified | none |
| AT4G18640 | GTLSSGVEIAVASTAIAESK | 8551700 | 86.539 | 0.0017969 | Unmodified | none |
| AT4G18640 | GTLSSGVEIAVASTAIAESK | 5195300 | 127.32 | 1,05E-01 | Unmodified | none |
| AT4G18640 | GTLSSGVEIAVASTAIAESK | 2435700 | 143.57 | 5,68E-03 | Unmodified | none |
| AT4G18640 | GTLSSGVEIAVASTAIAESK | 9517800 | 135.18 | 1,10E-02 | Unmodified | none |
| AT4G18640 | GTLSSGVEIAVASTAIAESK | 2033200 | 137.69 | 9,40E-03 | Unmodified | none |
| AT4G18640 | GTLSSGVEIAVASTAIAESK | 8151100 | 142.43 | 6,40E-03 | Unmodified | none |
| AT4G18640 | GTLSSGVEIAVASTAIAESK | 326220 | 66.636 | 0.034926 | Unmodified | none |
| AT4G18640 | GTLSSGVEIAVASTAIAESKEWTR | 7325200 | 52.769 | 0.027102 | Unmodified | none |
| AT4G18640 | GTLSSGVEIAVASTAIAESKEWTR | 565320 | 57.142 | 0.021168 | Unmodified | none |
| AT4G18640 | GTLSSGVEIAVASTAIAESKEWTR | 9035800 | 63.869 | 0.012969 | Unmodified | none |
| AT4G18640 | GTLSSGVEIAVASTAIAESKEWTR | 3297200 | 114.78 | 5,75E-03 | Unmodified | none |
| AT4G18640 | GTLSSGVEIAVASTAIAESKEWTR | 9086300 | 65.954 | 0.011517 | Unmodified | none |
| AT4G18640 | HVSGDLEQTSLLLPPEPEANVHSFGVLMLEIISGK | 3319500 | 56.099 | 0.00034618 | Unmodified | none |
| AT4G18640 | HVSGDLEQTSLLLPPEPEANVHSFGVLMLEIISGK | 4066800 | 40.064 | 0.018344 | Unmodified | none |
| AT4G18640 | HVSGDLEQTSLLLPPEPEANVHSFGVLMLEIISGK | 815510 | 53.987 | 0.00040575 | Unmodified | none |
| AT4G18640 | HVSGDLEQTSLLLPPEPEANVHSFGVLMLEIISGK | 1597800 | 38.381 | 0.021818 | Unmodified | none |
| AT4G18640 | KHVSGDLEQTSLLLPPEPEANVHSFGVLMLEIISGK | 10869000 | 60.562 | 0.00037829 | Unmodified | none |
| AT4G18640 | KHVSGDLEQTSLLLPPEPEANVHSFGVLMLEIISGK | 7088700 | 71.811 | 0.00010961 | Unmodified | none |
| AT4G18640 | KHVSGDLEQTSLLLPPEPEANVHSFGVLMLEIISGK | 2091100 | 37.63 | 0.033206 | Unmodified | none |
| AT4G18640 | KHVSGDLEQTSLLLPPEPEANVHSFGVLMLEIISGK | 2720600 | 63.576 | 0.00030631 | Unmodified | none |
| AT4G18640 | LNRSELETACEDFSNIIETFDGYTVYK | 1353300 | 79.663 | 0.00030756 | Unmodified | none |
| AT4G18640 | LSFSDEYGSIEQWASK | 550130 | 114.54 | 0.00089044 | Unmodified | none |
| AT4G18640 | LSFSDEYGSIEQWASK | 60919000 | 271.41 | 2,39E-50 | Unmodified | none |
| AT4G18640 | LSFSDEYGSIEQWASK | 45715000 | 215.93 | 9,69E-26 | Unmodified | none |
| AT4G18640 | LSFSDEYGSIEQWASK | 96546000 | 275.76 | 3,13E-62 | Unmodified | none |
| AT4G18640 | LSFSDEYGSIEQWASK | 114250000 | 190.18 | 6,38E-11 | Unmodified | none |
| AT4G18640 | LSFSDEYGSIEQWASK | 799700 | 167.18 | 2,02E-05 | Unmodified | none |
| AT4G18640 | LSFSDEYGSIEQWASK | 1141300 | 112.57 | 0.00091209 | Unmodified | none |
| AT4G18640 | MMVFEYAPNGTLFEHLHDKETEHLDWSAR | 7589200 | 55.163 | 0.0061086 | Unmodified | none |
| AT4G18640 | MMVFEYAPNGTLFEHLHDKETEHLDWSAR | 4074900 | 69.528 | 0.001025 | Unmodified | none |
| AT4G18640 | MMVFEYAPNGTLFEHLHDKETEHLDWSAR | 1042300 | 91.481 | 9,45E-01 | Oxidation (M) | none |
| AT4G18640 | NFVNLIGYCEEDDPFNR | 11426000 | 143.43 | 2,38E-02 | Unmodified | none |
| AT4G18640 | NFVNLIGYCEEDDPFNR | 10014000 | 114.21 | 0.00022971 | Unmodified | none |
| AT4G18640 | NFVNLIGYCEEDDPFNR | 19901000 | 105.02 | 0.00031534 | Unmodified | none |
| AT4G18640 | NFVNLIGYCEEDDPFNR | 18319000 | 165.66 | 3,85E-06 | Unmodified | none |
| AT4G22200 | N**S**IEAASNFVNR | 5016900 | 125.53 | 0.0023576 | Unmodified | none |
| AT4G22200 | N**S**IEAASNFVNR | 738440 | 109.15 | 0.0033923 | Unmodified | none |
| AT4G22200 | N**S**IEAASNFVNR | 27116000 | 188.15 | 1,54E-10 | Unmodified | none |
| AT4G22200 | N**S**IEAASNFVNR | 807580 | 52.101 | 0.032727 | Unmodified | none |
| AT4G22200 | N**S**IEAASNFVNR | 827690 | 91.202 | 0.010052 | Unmodified | none |
| AT4G22200 | N**S**IEAASNFVNR | 801810 | 176.99 | 2,25E-05 | Unmodified | none |
| AT4G22200 | N**S**IEAASNFVNR | 31630000 | 135.94 | 1,92E-11 | Unmodified | none |
| AT4G22200 | N**S**IEAASNFVNR | 624430 | 117.27 | 0.0028792 | Unmodified | none |
| AT4G22200 | N**S**IEAASNFVNR | 17745000 | 228.61 | 7,27E-27 | Unmodified | none |
| AT4G22200 | N**S**IEAASNFVNR | 600790 | 176.99 | 2,25E-05 | Unmodified | none |
| AT4G22200 | N**S**IEAASNFVNR | 24131000 | 162.31 | 5,00E-03 | Unmodified | none |
| AT4G22200 | N**S**IEAASNFVNR | 1444600 | 135.94 | 0.0012329 | Unmodified | none |
| AT4G22200 | N**S**IEAASNFVNR | 19266000 | 244.73 | 8,49E-29 | Unmodified | none |
| AT4G22200 | N**S**IEAASNFVNR | 49976000 | 244.73 | 8,49E-29 | Unmodified | none |
| AT4G18640 | QRPSMKDVAEQLK | 619240 | 116.9 | 0.011872 | Unmodified | none |
| AT4G18640 | QRPSMKDVAEQLK | 2638800 | 131.79 | 0.0055409 | Unmodified | none |
| AT4G18640 | QRPSMKDVAEQLK | 2379000 | 98.942 | 0.045154 | Unmodified | none |
| AT4G18640 | QRPSMKDVAEQLK | 5536000 | 139.46 | 0.0039387 | Unmodified | none |
| AT4G18640 | QRPSMKDVAEQLK | 604640 | 85.672 | 0.018805 | Unmodified | none |
| AT4G18640 | SELETACEDFSNIIETFDGYTVYK | 21461000 | 168.4 | 1,50E-13 | Unmodified | none |
| AT4G18640 | SELETACEDFSNIIETFDGYTVYK | 5413700 | 109.12 | 5,55E-05 | Unmodified | none |
| AT4G18640 | SELETACEDFSNIIETFDGYTVYK | 15499000 | 166.01 | 5,43E-13 | Unmodified | none |
| AT4G18640 | SELETACEDFSNIIETFDGYTVYK | 688780 | 83.826 | 2,99E-02 | Unmodified | none |
| AT4G18640 | SELETACEDFSNIIETFDGYTVYK | 756060 | 121.39 | 7,80E-05 | Unmodified | none |
| AT4G18640 | TFKEEELEVICDVIR | 2915100 | 152.25 | 6,30E-01 | Unmodified | none |
| AT4G18640 | TFKEEELEVICDVIR | 2266700 | 244.18 | 7,51E-27 | Unmodified | none |
| AT4G18640 | TFKEEELEVICDVIR | 2150500 | 167.18 | 2,22E-02 | Unmodified | none |
| AT4G18640 | TFKEEELEVICDVIRECLK | 2517900 | 66.606 | 0.024826 | Unmodified | none |
| AT4G18640 | TFKEEELEVICDVIRECLK | 390380 | 63.436 | 0.029254 | Unmodified | none |
| AT4G22200 | TMEFRN**S**IEAASNFVNR | 367210 | 98.227 | 0.00063169 | Unmodified | none |
| AT4G22200 | TMEFRN**S**IEAASNFVNR | 167610 | 62.055 | 0.025964 | Unmodified | none |
| AT4G18640 | VSEIPFNLEAR | 10558000 | 86.911 | 0.015952 | Unmodified | none |
| AT4G18640 | VSEIPFNLEAR | 27584000 | 84.892 | 0.019416 | Unmodified | none |
| AT4G18640 | VSEIPFNLEAR | 16217000 | 116.71 | 0.0016506 | Unmodified | none |
| AT4G18640 | VSEIPFNLEAR | 31302000 | 100.38 | 0.0065756 | Unmodified | none |
| AT4G18640 | YLEKDDLGEMIDPSLK | 21226000 | 139.46 | 0.00014354 | Unmodified | none |
| AT4G18640 | YLEKDDLGEMIDPSLK | 13156000 | 99.273 | 0.00083474 | Unmodified | none |
| AT4G18640 | YLEKDDLGEMIDPSLK | 36353000 | 132.5 | 0.00017765 | Unmodified | none |
| AT4G18640 | YLEKDDLGEMIDPSLK | 1486100 | 111.64 | 0.0002057 | Unmodified | none |
| AT4G18640 | YLEKDDLGEMIDPSLK | 44541000 | 142.4 | 0.00011794 | Unmodified | none |
| AT4G18640 | YLEKDDLGEMIDPSLK | 3344100 | 96.673 | 0.00052905 | Unmodified | none |
| AT4G18640 | YLEKDDLGEMIDPSLK | 3184400 | 88.319 | 0.012514 | Oxidation (M) | none |
| AT4G18640 | YLEKDDLGEMIDPSLK | 7134600 | 116.51 | 0.00045167 | Oxidation (M) | none |
| AT4G18640 | YLEKDDLGEMIDPSLK | 10815000 | 99.371 | 0.0052401 | Oxidation (M) | none |
| AT4G18640 | YLEKDDLGEMIDPSLK | 1987300 | 113.47 | 0.0036639 | Oxidation (M) | none |
| AT4G22200 | Y**S**YFWIR | 1151600 | 79.116 | 0.035827 | Unmodified | none |
| AT4G22200 | YSYFWIR | 2391500 | 94.006 | 0.0070045 | Unmodified | none |
| AT4G22200 | Y**S**YFWIR | 1691600 | 102.77 | 0.0036772 | Unmodified | none |
| AT4G22200 | Y**S**YFWIR | 1979000 | 107.32 | 0.0022289 | Unmodified | none |
| AT4G18640 | DDLGEMIDPSLK | 5695800 | 114.24 | 0.0014979 | Unmodified | triton |
| AT4G18640 | DDLGEMIDPSLK | 4549400 | 111.94 | 0.0019213 | Unmodified | triton |
| AT4G18640 | DVAEQLKQVINITPEK | 5660400 | 120.63 | 0.0022344 | Unmodified | triton |
| AT4G18640 | DVAEQLKQVINITPEK | 8600400 | 166.92 | 7,17E-06 | Unmodified | triton |
| AT4G18640 | DVAEQLKQVINITPEK | 8480200 | 127.3 | 0.00097284 | Unmodified | triton |
| AT4G18640 | GTLSSGVEIAVASTAIAESK | 11212000 | 159.01 | 4,12E-11 | Unmodified | triton |
| AT4G18640 | GTLSSGVEIAVASTAIAESK | 4147000 | 125.23 | 1,55E-02 | Unmodified | triton |
| AT4G18640 | GTLSSGVEIAVASTAIAESK | 15993000 | 144.4 | 2,65E-06 | Unmodified | triton |
| AT4G18640 | GTLSSGVEIAVASTAIAESK | 4220300 | 133.24 | 1,94E-05 | Unmodified | triton |
| AT4G18640 | GTLSSGVEIAVASTAIAESK | 10629000 | 81.807 | 0.0073971 | Unmodified | triton |
| AT4G18640 | GTLSSGVEIAVASTAIAESK | 784990 | 67.579 | 0.027487 | Unmodified | triton |
| AT4G18640 | GTLSSGVEIAVASTAIAESK | 1723100 | 117.37 | 7,10E-02 | Unmodified | triton |
| AT4G18640 | GTLSSGVEIAVASTAIAESKEWTR | 12326000 | 111.45 | 9,62E-02 | Unmodified | triton |
| AT4G18640 | GTLSSGVEIAVASTAIAESKEWTR | 16484000 | 45.659 | 0.042292 | Unmodified | triton |
| AT4G18640 | GTLSSGVEIAVASTAIAESKEWTR | 5850900 | 84.602 | 0.0088935 | Unmodified | triton |
| AT4G18640 | GTLSSGVEIAVASTAIAESKEWTR | 7607200 | 68.42 | 0.01549 | Unmodified | triton |
| AT4G18640 | GTLSSGVEIAVASTAIAESKEWTR | 6206800 | 90.378 | 0.0053102 | Unmodified | triton |
| AT4G18640 | HVSGDLEQTSLLLPPEPEANVHSFGVLMLEIISGK | 4242500 | 40.064 | 0.041078 | Unmodified | triton |
| AT4G18640 | HVSGDLEQTSLLLPPEPEANVHSFGVLMLEIISGK | 4148700 | 51.125 | 0.0024679 | Unmodified | triton |
| AT4G18640 | INHKNFVNLIGYCEEDDPFNR | 502980 | 87.062 | 0.015239 | Unmodified | triton |
| AT4G18640 | INHKNFVNLIGYCEEDDPFNR | 748370 | 37.884 | 0.043203 | Unmodified | triton |
| AT4G18640 | KHVSGDLEQTSLLLPPEPEANVHSFGVLMLEIISGK | 20636000 | 15.54 | 0.041142 | Unmodified | triton |
| AT4G18640 | KHVSGDLEQTSLLLPPEPEANVHSFGVLMLEIISGK | 13225000 | 40.864 | 0.024902 | Unmodified | triton |
| AT4G18640 | KHVSGDLEQTSLLLPPEPEANVHSFGVLMLEIISGK | 15835000 | 74.675 | 1,93E-03 | Unmodified | triton |

| AT4G18640 | KHVSGDLEQTSLLLPPEPEANVHSFGVLMLEIISGK | 1921900 | 21.958 | 0.038755 | Unmodified | triton |
| --- | --- | --- | --- | --- | --- | --- |
| AT4G18640 | KHVSGDLEQTSLLLPPEPEANVHSFGVLMLEIISGK | 950060 | 52.069 | 0.0030953 | Unmodified | triton |
| AT4G18640 | LSFSDEYGSIEQWASK | 126010000 | 188.9 | 4,63E-26 | Unmodified | triton |
| AT4G18640 | LSFSDEYGSIEQWASK | 68408000 | 197.73 | 1,83E-25 | Unmodified | triton |
| AT4G18640 | LSFSDEYGSIEQWASK | 397090 | 64.327 | 0.039895 | Unmodified | triton |
| AT4G18640 | LSFSDEYGSIEQWASK | 135240000 | 257.48 | 1,05E-60 | Unmodified | triton |
| AT4G18640 | LSFSDEYGSIEQWASK | 687710 | 123.76 | 1,67E-02 | Unmodified | triton |
| AT4G18640 | LSFSDEYGSIEQWASK | 706620 | 149.6 | 1,94E-10 | Unmodified | triton |
| AT4G18640 | LSFSDEYGSIEQWASK | 901770 | 149.6 | 1,94E-10 | Unmodified | triton |
| AT4G18640 | MMVFEYAPNGTLFEHLHDKETEHLDWSAR | 16442000 | 59.646 | 0.0035942 | Unmodified | triton |
| AT4G18640 | MMVFEYAPNGTLFEHLHDKETEHLDWSAR | 9483400 | 72.158 | 0.00034375 | Unmodified | triton |
| AT4G18640 | MMVFEYAPNGTLFEHLHDKETEHLDWSAR | 13548000 | 59.687 | 0.0035834 | Unmodified | triton |
| AT4G18640 | MMVFEYAPNGTLFEHLHDKETEHLDWSAR | 1244400 | 66.075 | 0.0019093 | Unmodified | triton |
| AT4G18640 | NFVNLIGYCEEDDPFNR | 10914000 | 182.91 | 3,28E-25 | Unmodified | triton |
| AT4G18640 | NFVNLIGYCEEDDPFNR | 13073000 | 118.05 | 1,97E-02 | Unmodified | triton |
| AT4G18640 | NFVNLIGYCEEDDPFNR | 28307000 | 204.9 | 2,24E-35 | Unmodified | triton |
| AT4G18640 | NFVNLIGYCEEDDPFNR | 19437000 | 91.518 | 0.0010085 | Unmodified | triton |
| AT4G22200 | N**S**IEAASNFVNR | 1319700 | 198.52 | 6,95E-25 | Unmodified | triton |
| AT4G22200 | N**S**IEAASNFVNR | 36210000 | 195.77 | 2,54E-18 | Unmodified | triton |
| AT4G22200 | N**S**IEAASNFVNR | 22702000 | 156 | 5,22E-05 | Unmodified | triton |
| AT4G22200 | N**S**IEAASNFVNR | 1622500 | 105.66 | 0.0031236 | Unmodified | triton |
| AT4G22200 | N**S**IEAASNFVNR | 43581000 | 105.66 | 5,96E-01 | Unmodified | triton |
| AT4G22200 | N**S**IEAASNFVNR | 90749000 | 221.09 | 1,94E-35 | Unmodified | triton |
| AT4G22200 | N**S**IEAASNFVNR | 1207300 | 99.669 | 0.0067288 | Unmodified | triton |
| AT4G22200 | N**S**IEAASNFVNR | 7709800 | 182.25 | 2,44E-16 | Unmodified | triton |
| AT4G22200 | N**S**IEAASNFVNR | 30795000 | 205.31 | 4,26E-26 | Unmodified | triton |
| AT4G22200 | N**S**IEAASNFVNR | 1004600 | 98.337 | 0.0078263 | Unmodified | triton |
| AT4G22200 | N**S**IEAASNFVNR | 5849700 | 187.62 | 1,57E-16 | Unmodified | triton |
| AT4G22200 | N**S**IEAASNFVNR | 18320000 | 215.02 | 4,22E-26 | Unmodified | triton |
| AT4G18640 | QRPSMKDVAEQLK | 428170 | 83.877 | 0.024089 | Oxidation (M) | triton |
| AT4G18640 | QRPSMKDVAEQLK | 1017300 | 104.41 | 0.011076 | Oxidation (M) | triton |
| AT4G18640 | QRPSMKDVAEQLK | 7802000 | 205.87 | 2,52E-10 | Unmodified | triton |
| AT4G18640 | QRPSMKDVAEQLK | 8866800 | 203.88 | 3,79E-10 | Unmodified | triton |
| AT4G18640 | QRPSMKDVAEQLK | 4607400 | 171.99 | 3,15E-03 | Unmodified | triton |
| AT4G18640 | QVINITPEK | 1357600 | 108.43 | 0.0053031 | Unmodified | triton |
| AT4G18640 | QVINITPEK | 1198200 | 98.044 | 0.01738 | Unmodified | triton |
| AT4G18640 | QVINITPEK | 4654400 | 104.42 | 0.0099639 | Unmodified | triton |
| AT4G18640 | SELETACEDFSNIIETFDGYTVYK | 13071000 | 162.72 | 1,11E-27 | Unmodified | triton |
| AT4G18640 | SELETACEDFSNIIETFDGYTVYK | 4157800 | 57.216 | 0.0022805 | Unmodified | triton |
| AT4G18640 | SELETACEDFSNIIETFDGYTVYK | 11786000 | 154.65 | 2,26E-18 | Unmodified | triton |
| AT4G18640 | SELETACEDFSNIIETFDGYTVYK | 3186800 | 76.049 | 7,52E-01 | Unmodified | triton |
| AT4G18640 | SELETACEDFSNIIETFDGYTVYK | 823750 | 80.868 | 4,57E-01 | Unmodified | triton |
| AT4G18640 | SELETACEDFSNIIETFDGYTVYK | 352100 | 74.251 | 8,84E-01 | Unmodified | triton |
| AT4G18640 | SELETACEDFSNIIETFDGYTVYK | 2187200 | 158.89 | 7,79E-19 | Unmodified | triton |
| AT4G18640 | TFKEEELEVICDVIR | 5141500 | 95.873 | 0.039035 | Unmodified | triton |
| AT4G18640 | TFKEEELEVICDVIR | 4644900 | 148.41 | 3,51E-02 | Unmodified | triton |
| AT4G18640 | TFKEEELEVICDVIR | 7335000 | 199.33 | 1,94E-16 | Unmodified | triton |
| AT4G18640 | TFKEEELEVICDVIR | 4723800 | 159.54 | 1,24E-04 | Unmodified | triton |
| AT4G18640 | TFKEEELEVICDVIRECLK | 511520 | 98.227 | 0.0024741 | Unmodified | triton |
| AT4G18640 | VSEIPFNLEAR | 34387000 | 110.31 | 0.0034443 | Unmodified | triton |
| AT4G18640 | VSEIPFNLEAR | 33257000 | 120.53 | 0.00082461 | Unmodified | triton |
| AT4G18640 | YLEKDDLGEMIDPSLK | 51249000 | 112.82 | 0.0035258 | Unmodified | triton |
| AT4G18640 | YLEKDDLGEMIDPSLK | 50966000 | 132.5 | 1,59E-01 | Unmodified | triton |
| AT4G18640 | YLEKDDLGEMIDPSLK | 64773000 | 120.9 | 0.0019977 | Unmodified | triton |
| AT4G18640 | YLEKDDLGEMIDPSLK | 4068100 | 106.76 | 0.0013035 | Unmodified | triton |
| AT4G18640 | YLEKDDLGEMIDPSLK | 51978000 | 115.57 | 0.0030053 | Unmodified | triton |
| AT4G18640 | YLEKDDLGEMIDPSLK | 1655000 | 93.237 | 0.0039077 | Unmodified | triton |
| AT4G18640 | YLEKDDLGEMIDPSLK | 11519000 | 96.143 | 0.010991 | Oxidation (M) | triton |
| AT4G18640 | YLEKDDLGEMIDPSLK | 17110000 | 123.08 | 0.00093363 | Oxidation (M) | triton |
| AT4G22200 | Y**S**YFWIR | 3714600 | 115.46 | 0.00034624 | Unmodified | triton |
| AT4G22200 | Y**S**YFWIR | 2359200 | 106.42 | 0.0055 | Unmodified | triton |
